# Predictive QSAR Models for the Toxicity of Disinfection Byproducts

**DOI:** 10.3390/molecules22101671

**Published:** 2017-10-09

**Authors:** Litang Qin, Xin Zhang, Yuhan Chen, Lingyun Mo, Honghu Zeng, Yanpeng Liang

**Affiliations:** 1 College of Environmental Science and Engineering, Guilin University of Technology, Guilin 541004, China; qinsar@163.com (L.Q.); zhangxxcmm@163.com (X.Z.); yu0002010@sina.cn (Y.C.); zenghonghu@glut.edu.cn (H.Z.); ypliang1980@163.com (Y.L.); 2 Guangxi Key Laboratory of Environmental Pollution Control Theory and Technology, Guilin University of Technology, Guilin 541004, China; 3 Collaborative Innovation Center for Water Pollution Control and Water Safety in Karst Area, Guilin University of Technology, Guilin 541004, China

**Keywords:** disinfection byproduct, QSAR, validation, toxicity, drinking water

## Abstract

Several hundred disinfection byproducts (DBPs) in drinking water have been identified, and are known to have potentially adverse health effects. There are toxicological data gaps for most DBPs, and the predictive method may provide an effective way to address this. The development of an in-silico model of toxicology endpoints of DBPs is rarely studied. The main aim of the present study is to develop predictive quantitative structure–activity relationship (QSAR) models for the reactive toxicities of 50 DBPs in the five bioassays of X-Microtox, GSH+, GSH−, DNA+ and DNA−. All-subset regression was used to select the optimal descriptors, and multiple linear-regression models were built. The developed QSAR models for five endpoints satisfied the internal and external validation criteria: coefficient of determination (*R*^2^) > 0.7, explained variance in leave-one-out prediction (*Q*^2^_LOO_) and in leave-many-out prediction (*Q*^2^_LMO_) > 0.6, variance explained in external prediction (*Q*^2^_F1_, *Q*^2^_F2_, and *Q*^2^_F3_) > 0.7, and concordance correlation coefficient (*CCC*) > 0.85. The application domains and the meaning of the selective descriptors for the QSAR models were discussed. The obtained QSAR models can be used in predicting the toxicities of the 50 DBPs.

## 1. Introduction

Disinfection byproducts (DBPs) have raised concerns since the first DBPs of trihalomethane (THM) compounds were identified in the 1970s [1]. The DBPs may result from reactions between disinfectants and dissolved organic matter present in source waters [2,3]. The THMs are the most common DBPs present in the typical chlorinated drinking water. Approximately 700 DBPs have been identified, as the increasingly employed disinfectants such as ozone, chlorine dioxide and chloramines in drinking water result in reactivity with organic compounds [2]. Research has demonstrated that several known DBPs are considered to be potent cytotoxins, genotoxins and carcinogens [4], which indicates that many DBPs may exert more toxicity to humans than THMs [3]. However, there are toxicological data gaps for most of the DBPs, and more in vitro bioassays or chronic in vivo bioassays need to be carried out. Compared with the experimental test for the toxicological data, the in-silico approach provides an effective way for predicting the toxicities of chemicals. Quantitative structure–activity relationship (QSAR) seems to be a useful method to study the toxicities of DBPs.

The QSAR method provides a promising, faster way of predicting the activity of chemicals using the structural information of the compound. A limited number of QSARs have been proposed for DBP studies. A QSAR for benchmark concentrations of cranial neural tube dysmorphogenesis was established for 10 halogenated derivatives (HAAs) [5]. The mutagenicity in the *Salmonlla typhimurium* strain TA100 of 42 HAAs was predicted by a QSAR model based on geometrical-, radial distribution function (RDF)-, weighted holistic invariant molecular (WHIM)-, eigenvalue- and 2D-autocorrelation-based methods, as well as information descriptors [6]. The eight-variable model was internally validated by leave-one-out (LOO) cross-validation, the bootstrapping test and Y-scrambling. Tang and Wang [7] developed a QSAR model for finding the energy of the highest occupied molecular orbital (E_HOMO_) of 36 DBPs, and the model was validated by LOO cross-validation and K-fold cross-validation. An empirical QSAR model based on liposome–water partition coefficients (logK_lipw_) was proposed for the 50% effective concentration (EC_50_) of five DBPs (1,1-dichloroethene, bromoethane, chloroform, dichloromethane and bromoform) on *Vibrio fischeri* [8]. Another empirical model was built for the first-order rate constants of photodegradation of six iodinated trihalomethanes (ITHMs) and three brominated THMs (BTHMs) [9]. Yang and Zhang [10] predicted the developmental toxicity of 19 DBPs to *Platynereis dumerilii* embryos using an oil–water partition coefficient (logP), protein kinase A (pK(a)), E_HOMO_ and lowest unoccupied molecular orbital energy (E_LUMO_). All the aforementioned QSAR models for DBPs lack both strictly internal and external validation, which may not guarantee the real predictive ability of the models. Only two studies [8,10] were related to the toxicities of DBPs with a limited number of compounds (no more than 19 samples). The main reasons for the lack of QSAR study on DBPs is that only a small fraction of the DBPs identified (out of a total of ~700) have been tested for toxicity so far. It is implied that QSAR techniques remain underutilized by DBP researchers [2].

In the present study, we aim to develop QSAR models using multiple linear regression (MLR) to predict five toxicity endpoints of DBPs. The developed QSAR models were strictly internally validated by LOO and leave-many-out (LMO) cross-validation and Y-scrambling, and externally validated by several metrics, including variance explained in external prediction *Q*^2^_F1_ [11], *Q*^2^_F2_ [12], and *Q*^2^_F3_ [13], concordance correlation coefficient (*CCC*) [14,15], and $r_{m}^{2}$ metrics based on the correlation of the observed and predicted response data with and without the intercept [16,17], and the criteria recommended by Golbraikh and Tropsha [18]. 

## 2. Materials and Methods

### 2.1. Experimental Toxicity Data

The five endpoints of 50 drinking water DBPs are precisely explained (Table 1). Experimental reactive toxicity data for five endpoints (X-Microtox, GSH+, GSH−, DNA+ and DNA−) of 50 drinking water DBPs were obtained from the literature (Table 2) [4]. The 50 DBPs comprise a wide range of different chemical groups, 47 commonly detected drinking water DBPs together with 1,1-dichloroethene (1,1-DCE), dichloromethane (DCM) and bromochloromethane. The negative logarithm observed effect concentrations (pEC_50_ for Microtox and pEC_IR1.5_ for the other assays, M (mol/L)) are listed in Table 2.

### 2.2. Molecular Structure Descriptors

The molecular structure descriptors of the chemicals were calculated by the Dragon software (version 6.0, Talete SRL, Milano, Italy). The original descriptors generated from the Dragon software were refined by the following principles [19,20]: (1) the descriptors with standard deviation less than 0.0001 were excluded; (2) the descriptors with at least one missing value were deleted; (3) the descriptors with (abs)pair correlation larger than or equal to 0.8 were excluded; and (4) the descriptors that Pearson correlation coefficients (|*r*|) between descriptors and toxicities of DBPs lower than 0.3 were deleted. The remaining descriptors were used for the further analysis. 

### 2.3. Data Splits and Model Development

The whole dataset was randomly split into several training and test sets. It was recommended that analysis of the models should be obtained from various splits into the training set and test set [21]. For each toxicity endpoint of DBPs, we randomly split the whole dataset into five training sets and five test sets.

All-subset regression for the whole dataset was performed with the QSARINS software [22,23]. The four-variable multiple linear regression (MLR) models with the highest coefficients of determination (*R*^2^) and explained variance in leave-one-out (*Q*^2^_LOO_) prediction were selected for the whole dataset. The MLR models for the training sets based on the same descriptors derived from the whole dataset were developed, and the test sets were used to validate the external predictive abilities of the models.

### 2.4. Model Validation

The statistical parameters for modeling, internal and external validation metrics were adopted to evaluate the fit, stability and predicative power of the QSAR model. The quality parameters include *R*^2^, adjusted coefficient of determination ($R_{\mathrm{adj}}^{2}$), root mean square error in fitting (*RMSE*_tr_) and *F*-value (*F*). The internal validations were performed by the LOO and LMO cross-validation (*Q*^2^_LOO_ and *Q*^2^_LMO_) and the Y-scrambling test (*R*^2^_Yscr_ and *Q*^2^_Yscr_). The external validation was evaluated by a test set. The parameters *Q*^2^_F1_ [11], *Q*^2^_F2_ [12], *Q*^2^_F3_ [13], *CCC* [14,15], average of $r_{m}^{2}$ (${\bar{r}}_{m}^{2}$) and the difference between $r_{m}^{2}$ ($\Delta r_{m}^{2}$) [16,17] were used as the measures of the predictive power of a QSAR model. The proposed parameters by Golbraikh and Tropsha were also applied for the external validation criteria [18]: slope of the regression line over external data (*k* and *k’*), coefficients of determination between predicted and observed activities ($R_{0}^{2}$) and coefficients of determination between observed and predicted activities (${R^{\prime }}_{0}^{2}$).

The validation criteria thresholds for the parameters mentioned above are: (1) *R*^2^ > 0.7, *Q*^2^_LOO_ and *Q*^2^_LMO_ > 0.6, *Q*^2^_F1_, *Q*^2^_F2_ and *Q*^2^_F3_ > 0.7, difference between *R*^2^ and *Q*^2^_LOO_ smaller than 0.1 [15]; (2) ${\bar{r}}_{m}^{2}$ > 0.65; (3) *CCC* > 0.85 [15]; and (4) criteria recommended by Golbraikh and Tropsha [18]: $(R^{2}-R_{0}^{2})/R^{2}<0.1$ or $(R^{2}-{R^{\prime }}_{0}^{2})/R^{2}<0.1$, 0.85 ≤ *k* ≤ 1.15 or 0.85 ≤ *k*’ ≤ 1.15.

The descriptors included in the whole dataset, training set and the test set should satisfy the following conditions [24]: (1) the Pearson correlation coefficients for the complete (*r*_c_), training (*r*_t_) and test (*r*_e_) sets are equal to or greater than 0.3: |*r*_c_| and |*r*_t_| ≥ 0.3; (2) the normalized regression coefficient of the descriptor for the complete (*β*_c_) and the training (*β*_t_) sets are equal to or greater than 0.001: |*β*_c_| and |*β*_t_| ≥ 0.001; and (3) absence of the sign-change problem: sign(*r*_c_) = sign(*r*_t_) = sign(*r*_e_); sign(*r*_c_) = sign(*β*_c_) = sign(*β*_t_)

Models that have acceptable validation criteria thresholds for all conditions were considered as the final models. These models are robust and able to make good internal and external predictions.

### 2.5. Applicability Domain

The application domain of the QSAR model was defined by the leverage approach from the hat matrix (*h*_i_ in Equation (1)), which is calculated from the descriptors of chemicals [19,25], and by identification of chemicals with LOO cross-validated standardized residuals greater than 2.0 standard deviation units. An outlier in the QSAR model is defined as *h_i_* value larger than the warning leverage h* and LOO standardized residuals greater than 2.0, which is graphically depicted in the Williams plot. The warning leverage h^*^ is fixed at (3*k*)/*n*, where *k* is the number of model parameters and *n* is the number of the objects used to calculate the model.

(1) $$h_{i}=x_{i}^{T}{\left(X^{T}X\right)}^{-1}x_{i}\;(i=1,\ldots ,n)$$

where *x_i_* is the descriptor row vector of the query compound; *X* is the *n* × *k* matrix of *k* model descriptor values for *n* training set compounds and the superscript *T* refers to the transpose of the matrix/vector.

## 3. Results and Discussion

### 3.1. Selected Descriptors

For each five endpoints of X-Microtox, GSH+, GSH−, DNA+ and DNA− of the selected drinking-water DBPs, four descriptors were selected by all-subset regression for the whole dataset, which was performed by the QSARINS software [22,23]. The selected descriptors for X-Microtox were the spectral diameter from Burden matrix weighted by mass (SpDiam_B(m)), average vertex sum from Burden matrix weighted by van der Waals volume (AVS_B(v)), eigenvalue no. 5 from augmented edge adjacency mat weighted by dipole moment (Eig05_AEA(dm)) and sum of ddsN E-states (SddsN). For the endpoints of GSH+ and GSH−, the four selected descriptors were percentage of C atoms (C%), SpDiam_B(m), P_VSA-like on LogP bin 8 (P_VSA_LogP_8) and sum of topological distances between N..Br (T(N..Br)). The selected descriptors for DNA+ were P_VSA-like on LogP, bin 7 (P_VSA_LogP_7), signal 04/weighted by I-state (Mor04s), T(N..Br) and sum of topological distances between N..I (T(N..I)). For the endpoints of DNA−, the four selected descriptors were sum of atomic van der Waals volumes (Sv), P_VSA_LogP_7, signal 03/weighted by I-state (Mor03s) and T(N..I). There was no high correlation between the selected descriptors, and these descriptors were used as inputs for the training set.

### 3.2. Models Development and Validation

The whole dataset for each endpoint was randomly split into training and test sets by five iterations (splits 1–5) for the same size of training and test sets. Of the chemicals in the dataset, 80% were selected for the training set and the remaining 20% were considered as the test set. Five QSAR models based on the same size of training sets were built for five endpoints of X-Microtox, GSH+, GSH−, DNA+ and DNA−. The statistical parameters of modeling, internal and external validations were calculated for each model. We have examined five splits into the training and test sets. The realistic reliability of the QSAR model was estimated by the result of the analysis of five splits into the training and test sets. The statistical characteristics of QSAR models of five splits for five endpoints are given in Supplementary data. It can be found that all QSAR models presented high predictive power, as those models satisfy the internal and external validation criteria: *R*^2^ > 0.7, *Q*^2^_LOO_ and *Q*^2^_LMO_ > 0.6, *Q*^2^_F1_, *Q*^2^_F2_ and *Q*^2^_F3_ > 0.7, *CCC* > 0.85, and ${\bar{r}}_{m}^{2}$ > 0.65.

In order to validate whether the descriptors presented in the QSAR models were real or not before model validation and interpretation, we checked the sign-change-problem correlation coefficients and regression coefficients of a descriptor in the MLR model regressions, before and after the data split [24]. It was found that all descriptors in the five QSAR models satisfy the conditions [24]: |*r*_c_| and |*r*_t_| ≥ 0.3, |*β*_c_| and |*β*_t_| ≥ 0.001, sign(*r*_c_) = sign(*r*_t_) = sign(*r*_e_), and sign(*r*_c_) = sign(*β*_c_) = sign(*β*_t_). Thus, the selected descriptors are considered to be real variables.

**Figure 1:**
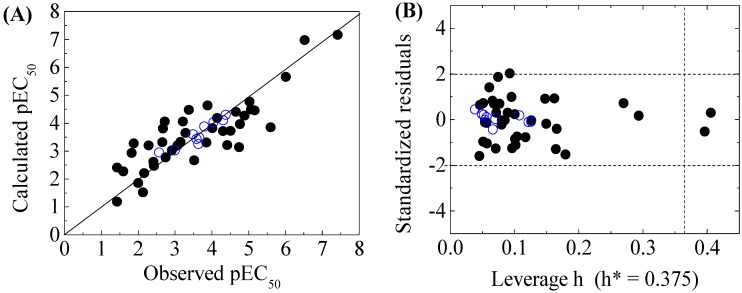
Scatter plot of observed versus calculated pEC_50_ (**A**), and the Williams plot of the final model (**B**) for 50 disinfection byproducts to X-Microtox. “●”: training set, “○”: test set. h*( warning leverage).

**Figure 2:**
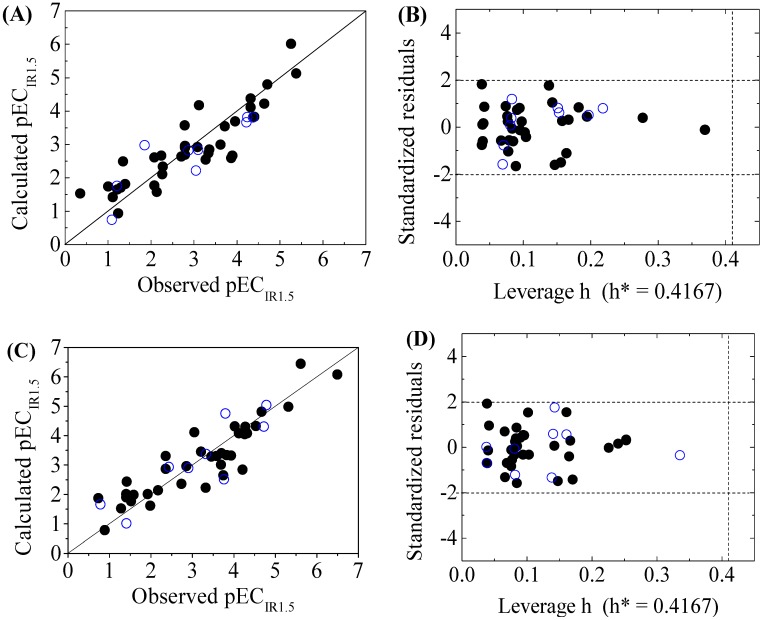
Scatter plot of observed versus calculated pEC_IR1.5_ ((**A**) for GSH+ and (**C**) for GSH− ) and the Williams plot ((**B**) for GSH+ and (**D**) for GSH− ) of the final model for 45 disinfection byproducts. “●”: training set, “○”: test set.

**Figure 3:**
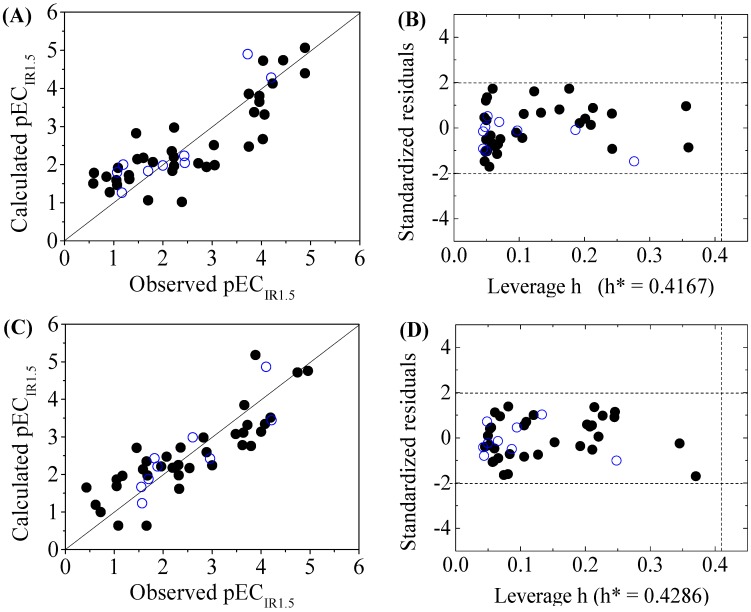
Scatter plot of observed versus calculated pEC_IR1.5_ ((**A**) for DNA+ and (**C**) for DNA−) and the Williams plot ((**B**) for DNA+ and (**D**) for DNA−) of the final model for 45 disinfection byproducts. “●”: training set, “○”: test set.

The four-variable QSAR models for the first split and its statistical parameters for five toxicity endpoints are listed in Table 3. All five QSAR models for toxicity bioassays of X-Microtox, GSH+, GSH−, DNA+ and DNA− are satisfactory, according to all conditions of *R*^2^ > 0.7, *Q*^2^_LOO_ and *Q*^2^_LMO_ > 0.6, *Q*^2^_F1_, *Q*^2^_F2_ and *Q*^2^_F3_ > 0.7, *CCC* > 0.85 [15]; ${\bar{r}}_{m}^{2}$ > 0.65; $(R^{2}-R_{0}^{2})/R^{2}<0.1$ or $(R^{2}-{R^{\prime }}_{0}^{2})/R^{2}<0.1$, and 0.85 ≤ *k* ≤ 1.15 or 0.85 ≤ *k*’ ≤ 1.15 [18]. Figure 1, Figure 2 and Figure 3 present the correlations between experimental and calculated –logEC_50_ or –logEC_IR1.5_ (pEC_50_ or pEC_IR1.5_) values for the five models. The pEC_50_ or pEC_IR1.5_ values calculated from the QSAR models are listed in Table 2.

### 3.3. Domain of Applicability

The criteria for an outlier are expressed as *h_i_* > h^*^ and LOO standardized residuals greater than 2.0. For the first split (split 1), the Williams plot of the five QSAR models for toxicity bioassays of X-Microtox, GSH+, GSH−, DNA+ and DNA− are shown in Figure 1, Figure 2 and Figure 3. All 50 DBPs in the training and test sets satisfy the outlier criteria, and the QSAR models lead to reliably predicted data. The outlier was also examined for the splits 2–5 (the statistical parameters are listed in Supplementary data). There were no outliers in the training and test sets.

### 3.4. Explanation of Descriptors 

The five developed QSAR models allow for mechanical interpretation of the toxicities of DBPs to X-Microtox, GSH+, GSH−, DNA+ and DNA−. Four descriptors for five models selected by stepwise MLR helped to improve the understanding of DBPs. A total of 12 descriptors were included in five four-variable models. The selected descriptors are SpDiam_B(m), AVS_B(v), Eig05_AEA(dm), SddsN, Sv, C%, P_VSA_LogP_7, P_VSA_LogP_8, Mor03s, Mor04s, T(N..Br) and T(N..I). These structural features are related to DBP toxicity. The positive values of the regression coefficients indicate increasing toxicity with increasing descriptor values, while the negative values indicate decreasing toxicity with increasing descriptor values. The 12 descriptors belong to different groups of descriptors: 2D matrix-based descriptors (SpDiam_B(m) and AVS_B(v)), edge adjacency indices (Eig05_AEA(dm)), atom-type E-state indices (SddsN), constitutional indices (Sv and C%), P_VSA-like descriptors (P_VSA_LogP_7 and P_VSA_LogP_8), 3D-MoRSE descriptors (Mor03s and Mor04s) and 2D atom pairs (T(N..Br) and T(N..I)).

For the QSAR model based on the toxicity of the X-Microtox bioassay, the standard regression coefficients of AVS_B(v) and SddsN were higher than the other two descriptors. AVS_B(v) and SddsN were the main factors affecting the toxicity of DBPs to X-Microtox. The descriptor SddsN indicated that −N(=)= (nitro) (where “=” represents a double bond and “−” represents a single bond) is one of the main factors that affected the toxicity of DBPs to X-Microtox. For the toxicities of DBPs toward GSH+ and GSH−, the same descriptors were selected in the QSAR models, which indicates the similar toxicity mechanism of the two endpoints. SpDiam_B(m) and T(N..Br) were the main positive contributors to the toxicity, as their standard regression coefficients were higher than C% and P_VSA_LogP_8. T(N..Br), the heteroatom between N and Br, was one of the main factors affecting the toxicity of DBPs toward GSH+ and GSH−. There were two descriptors (P_VSA_LogP_7 and T(N..I)) in the QSAR model for DNA+ and DNA−, where P_VSA_LogP_7 made a positive contribution to toxicity while T(N..I) made a negative contribution to toxicity. T(N..I), the heteroatom between N and I, was one of the main factors affecting the toxicity of DBPs toward DNA+ and DNA−. 

## 4. Conclusions

All five considered QSAR models, resulting from the random split of the whole dataset intro training and test sets, satisfied the validation criteria. The application domain was clearly defined and the mechanism was interpreted. The reliability of the five QSAR models met the Organization for Economic Co-operation and Development (OECD) principles [26]: (1) a defined endpoint; (2) an unambiguous algorithm; (3) a defined domain of applicability; (4) appropriate measures of goodness-of-fit, robustness and predictivity; and (5) a mechanistic interpretation, if possible.

The QSAR method was used to develop several predictive models for the toxicities of DBPs toward X-Microtox, GSH+, GSH−, DNA+, and DNA−. Using the selected descriptors, which can be easily generated from the Dragon software, all the developed QSAR models with a good predictive performance were used for estimating toxicities of DBPs. It is expected that the proposed QSAR models could be used to predict the toxicities of DBPs. 

## Figures and Tables

**Table 1:** The five endpoints of the 50 drinking water disinfection byproducts (DBPs).

Bioassay	Test Species (Strain/Cell Line) ^a^	Endpoint	Detected Signal
Microtox	*Aliivibrio fischeri*	Cytotoxicity	Bioluminescence as indicator for cell viability
*E. coli* ± GSH	*Escherichia coli* MJF335 (GSH−) and MJF276 (GSH+)	Interaction with proteins/peptides	OD at 600 nm as indicator for cell density and descriptor of cell growth
*E. coli* ± DNA	*Escherichia coli* MV4108 (DNA−) and MV1161 (DNA+)	Interaction with DNA	OD at 600 nm as indicator for cell density and descriptor of cell growth

a: GSH+: EC_50_ of *E. coli* strain; MJF276: capable to produce glutathione (GSH); GSH−: EC_50_ of *E. coli* strain; MJF335: not capable to produce GSH and hence more susceptible to compounds which react with proteins (i.e., soft electrophiles); DNA+: EC_50_ of *E. coli* strain; MV1161: capable of repairing DNA damage; DNA−: EC_50_ of *E. coli* strain; MV4108: not capable of repairing DNA damage and hence more susceptible to compounds which react with DNA (i.e., hard electrophiles).

**Table 2:** Observed and calculated effect concentrations (pEC_50_ (negative logarithm of 50% effective concentration) for X-Microtox and pEC_IR1.5_ (negative logarithm of 1.5 induction ratio effective concentration) for the other assays, mol/L) of disinfection byproducts.

No.	Name	X-Microtox		GSH+		GSH−		DNA+		DNA−	
		Observed pEC_50_	Calculated pEC_50_	Observed pEC_IR1.5_	Calculated pEC_IR1.5_	Observed pEC_IR1.5_	Calculated pEC_IR1.5_	Observed pEC_IR1.5_	Calculated pEC_IR1.5_	Observed pEC_IR1.5_	Calculated pEC_IR1.5_
	**Halomethanes**										
1	1,1-dichloroethene	3.1549	3.3214	1.3516	2.4847	1.4145	2.4357	1.0706 *	1.7985 *	0.4318	1.6512
2	dichloromethane	2.2840	3.1963	1.0888 *	0.7391 *	0.8861	0.7818	0.8539	1.6828	0.7212	0.9997
3	bromochloromethane	5.0706	4.4813	1.2182	1.6474	0.7328	1.8741	0.6021	1.7808	-	-
4	chloroform	2.4318	2.4675	1.2366	0.9330	1.4089 *	1.0149 *	1.1675 *	1.2621 *	1.0809	0.6385
5	bromodichloromethane	5.0269	4.7723	1.2111 *	1.7614 *	1.4034	1.9032	1.3188	1.6196	1.5686 *	1.2324 *
6	bromoform	3.6383 *	3.2549 *	1.0066	1.7407	1.5850	1.9863	1.0862	1.9166	1.1675	1.9629
7	dibromochloromethane	3.0000 *	3.0193 *	2.0809	1.7617	1.9208	2.0116	1.6990 *	1.8321 *	1.5528 *	1.6647 *
8	dichloroiodomethane	3.4949 *	3.5960 *	2.7959	2.9585	3.2076	3.4508	2.1938	1.8353	2.3279	1.6163
9	bromochloroiodomethane	1.6021	2.2717	2.7959	2.8967	3.3279 *	3.3766 *	2.2291	2.9723	2.3188	1.9777
10	dibromoiodomethane	4.0506 *	4.0353 *	2.8697 *	2.8209 *	3.4559	3.2854	2.0000 *	1.9755 *	1.9586	2.2126
11	chlorodiiodomethane	4.6576	4.4046	3.0809	2.9161	3.6990	3.3999	2.4437 *	2.0457 *	2.3098	2.2495
12	bromodiiodomethane	5.6021	3.8512	3.0969 *	2.8369 *	3.5850	3.3046	3.0506	1.9836	2.9586 *	2.4210 *
13	triiodomethane	2.4202	2.6149	3.3615	2.8486	3.9337	3.3188	2.8861	1.9398	2.8861	2.5894
	**Halonitromethanes**										
14	trichloronitromethane	4.3098	3.7132	4.6383	4.2152	5.3143	4.9812	4.2007 *	4.2819 *	4.0809	3.3428
15	tribromonitromethane	2.7447	2.7705	5.3820	5.1283	6.4949	6.0793	4.8861	5.0640	4.7447	4.7139
	**Haloacetonitriles**										
16	dichloroacetonitrile	4.5086	3.7184	3.2757	2.5481	3.7632 *	2.5119 *	3.0362	2.5123	2.8239	2.9808
17	trichloroacetonitrile	4.8861	4.2672	3.8979	2.6617	3.7447	2.6486	3.7447	3.8560	3.4815	3.0753
18	bromochloroacetonitrile	4.0132	3.8159	4.3188	4.1067	4.2757	4.2982	3.8539	3.3736	3.7212	3.3159
19	dibromoacetonitrile	4.7696	3.9655	4.7100	4.7981	4.7825 *	5.0416 *	4.2291	4.1282	4.1938	3.5113
	**Haloketones**										
20	1,1-dichloropropanone	2.7212	4.0555	3.0506 *	2.2187 *	3.3188	2.2263	2.4318 *	2.2303 *	2.3565	2.7129
21	1,1,1-trichloropropanone	3.6576 *	3.4857 *	2.2803	2.3311	2.7364	2.3615	2.3872	1.0225	3.0000	2.2423
	**Haloacetic acids**										
22	chloroacetic acid	6.0088	5.6592	2.1367	1.5737	1.9851	1.6179	1.6990	1.0652	1.6576	2.3448
23	bromoacetic acid	1.8861	3.2782	3.8697	2.5921	4.2111	2.8428	4.0655	3.3127	4.0000	3.1346
24	iodoacetic acid	2.6778	3.8068	4.3768 *	3.8079 *	4.7212 *	4.305 *	3.7447	2.4730	3.6576	3.8483
25	dichloroacetic acid	3.2147	4.0622	1.2967	1.6975	1.5229	1.7669	0.9208	1.2745	0.6198	1.1912
26	bromochloroacetic acid	5.1612	4.4508	2.0783	2.6122	2.3565	2.8669	1.1938 *	1.9983 *	1.6990 *	1.8777 *
27	dibromoacetic acid	4.1487	4.1865	2.2403	2.6637	2.4318 *	2.9289 *	1.6021	2.1776	1.8861 *	2.2108 *
28	chloroiodoacetic acid	1.8239	2.9267	4.4034	3.8242	4.5302	4.3246	4.0362	4.7239	4.1024 *	4.8634 *
29	bromoiodoacetic acid	3.7959 *	3.8762 *	4.2403 *	3.8168 *	4.0200	4.3157	3.7212 *	4.8968 *	3.8861	5.1786
30	trichloroacetic acid	3.2924	3.6489	1.4034	1.8108	1.4034	2.0112	1.0555	1.5905	1.0506	1.6881
31	bromodichloroacetic acid	1.4318	2.4029	2.7100	2.6367	2.9031 *	2.8964 *	1.7959	2.0658	1.6576	0.6340
32	dibromochloroacetic acid	3.5229	2.6718	2.7959	2.6882	2.8539	2.9584	1.4815	2.1417	1.4559	2.7074
33	tribromoacetic acid	4.4202	3.2073	3.3372	2.7322	3.6882	3.0113	2.1805	2.3490	2.6021 *	2.9859 *
	**Haloacetaldehyde**										
34	chloral hydrate	2.1675	2.2067	2.2636	2.1046	2.1707	2.1359	1.3098	1.7185	1.6778	1.9750
	**Haloacetamides**										
35	dichloracetamide	6.5229	6.9769	1.1135	1.4161	1.2798	1.5222	0.5850	1.5056	1.0506	1.6881
36	bromochloroacetamide	2.5686 *	2.9516 *	1.8539 *	2.9712 *	2.3565	3.3043	1.4559	2.8215	1.8239 *	2.4295 *
37	dibromoacetamide	3.0706	3.2043	4.2218 *	3.6626 *	4.2596	4.0477	3.9586	3.6467	3.6198	2.7807
38	chloroiodoacetamide	2.6576	3.3142	3.7212	3.5437	4.1192	4.0810	2.7212	2.0333	2.5376	2.1670
39	bromoiodoacetamide	3.3768	4.4729	3.1163	4.1762	3.7959 *	4.7536 *	2.2291	1.9651	2.0706	2.4718
40	diiodoacetamide	1.4318	1.1768	2.7825	3.5724	3.0482	4.1155	2.2218	2.1941	2.1938	2.1785
41	trichloroacetamide	2.0000	1.8559	0.3565	1.5288	0.7825 *	1.6577 *	1.0706	1.4651	1.5850	2.1329
42	bromodichloroacetamide	4.3098 *	4.099 *	3.6198	2.9951	3.8239	3.3331	4.0315	2.6693	3.7959	2.7569
43	dibromochloroacetamide	4.3768 *	4.2953 *	3.9566	3.6865	4.3188	4.0764	3.9586	3.8000	3.6383	3.1107
44	tribromoacetamide	2.1308	1.5148	4.3233	4.3703	4.6676	4.8106	4.4437	4.7359	4.2147 *	3.4421 *
	**Nitrosamines**										
45	n-nitrosodimethylamine	2.9208	3.0246	-	-	-	-	-	-	-	-
46	n-nitrosodiethylamine	7.4202	7.1686	-	-	-	-	-	-	-	-
47	n-nitrosopiperidine	3.8861	4.6292	-	-	-	-	-	-	-	-
48	n-nitrosomorpholine	3.8539	3.3096	-	-	-	-	-	-	-	-
49	nitrosodi-n-butylamine	3.5850 *	3.4285 *	-	-	-	-	-	-	-	-
	**Furanone**										
50	3-chloro-4-(dichloromethyl)-5-	4.7447	3.1323	5.2596	6.0139	5.6108	6.4454	4.8861	4.3949	4.9586	4.7578

* The chemical included in the test set.

**Table 3:** QSAR(quantitative structure–activity relationship) model and statistical parameters for five endpoints of disinfection byproduct (training set = 80% of whole dataset, test set = 20% of whole dataset).

Endpoint	Equation ^a^	Modeling ^b^	Internal Validation ^c^	External Validation ^d^	Golbraikh & Tropsha ^e^
X-Microtox	pEC_50_ = −11.8502 + 0.1230 SpDiam_B(m) + 4.9744 AVS_B(v) + 0.8805 Eig05_AEA(dm) − 3.3986 SddsN	*n*_tr_ = 40, *R*^2^ = 0.7152, $R_{\mathrm{adj}}^{2}$ = 0.6826, *RMSE*_tr_ = 0.7682, *F* = 21.9717	$Q_{LOO}^{2}$ = 0.6374, *RMSE*_cv_ = 0.8668, $Q_{LMO}^{2}$ = 0.6216, $R_{Y\mathrm{scr}}^{2}$ = 0.1034, $Q_{Y\mathrm{scr}}^{2}$ = −0.2452	*n*_test_ = 10, *RMSE*_ext_ = 0.2040, $R_{\mathrm{ext}}^{2}$ = 0.8660, $Q_{F1}^{2}$ = 0.8508, $Q_{F2}^{2}$ = 0.8496, $Q_{F3}^{2}$ = 0.9799, *CCC* = 0.9115 ${\bar{r}}_{m}^{2}$ = 0.7185, $\Delta r_{m}^{2}$ = 0.1439	*k* = 1.0136, *k*’ = 0.9837, $R_{0}^{2}$ = 0.8018, ${R^{\prime }}_{0}^{2}$ = 0.8584
GSH+	pEC_IR1.5_ = −2.4744 + 0.1022C% + 0.3184SpDiam_B(m) + 0.0725 P_VSA_LogP_8+ 0.2132 T(N..Br)	*n*_tr_ = 36, *R*^2^ = 0.7837, $R_{\mathrm{adj}}^{2}$ = 0.7558, *RMSE*_tr_ = 0.5927, *F* = 28.0843	$Q_{LOO}^{2}$ = 0.6956, *RMSE*_cv_ = 0.7032, $Q_{LMO}^{2}$ = 0.6644, $R_{Y\mathrm{scr}}^{2}$ = 0.1121, $Q_{Y\mathrm{scr}}^{2}$ = −0.2323	*n*_test_ = 9, *RMSE*_ext_ = 0.6010, $R_{\mathrm{ext}}^{2}$ = 0.7715, $Q_{F1}^{2}$ = 0.7502, $Q_{F2}^{2}$ = 0.7502, $Q_{F3}^{2}$ = 0.7776, *CCC* = 0.8500, ${\bar{r}}_{m}^{2}$ = 0.6558, $\Delta r_{m}^{2}$ = 0.1915	*k* = 1.0596, *k*’ = 0.9119, $R_{0}^{2}$ = 0.6964, ${R^{\prime }}_{0}^{2}$ = 0.7709
GSH-	pEC_IR1.5_ = −2.4133 + 0.0894 C% + 0.3829SpDiam_B(m) + 0.0835 P_VSA_LogP_8 + 0.2270 T(N..Br)	*n*_tr_ = 36, *R*^2^ = 0.8166, $R_{\mathrm{adj}}^{2}$ = 0.7929, *RMSE*_tr_ = 0.5936, *F* = 34.5096	$Q_{LOO}^{2}$ = 0.7332, *RMSE*_cv_ = 0.7160, $Q_{LMO}^{2}$ = 0.6634, $R_{Y\mathrm{scr}}^{2}$ = 0.1140, $Q_{Y\mathrm{scr}}^{2}$ = −0.2349	*n*_test_ = 9, *RMSE*_ext_ = 0.6578, $R_{\mathrm{ext}}^{2}$ = 0.7593, $Q_{F1}^{2}$ = 0.7436, $Q_{F2}^{2}$ = 0.7430, $Q_{F3}^{2}$ = 0.7748, *CCC* = 0.8703, ${\bar{r}}_{m}^{2}$ = 0.6688, $\Delta r_{m}^{2}$ = 0.0426	*k* = 0.9659, *k*’ = 0.9969, $R_{0}^{2}$ = 0.7376, ${R^{\prime }}_{0}^{2}$ = 0.7510
DNA+	pEC_IR1.5_ = 1.8732 + 0.0493 P_VSA_LogP_7 − 0.2258 Mor04s + 0.2798 T(N..Br) − 0.8971 T(N..I)	*n*_tr_ = 36, *R*^2^ = 0.7019, $R_{\mathrm{adj}}^{2}$ = 0.6635, *RMSE*_tr_ = 0.7113, *F* = 18.2520	$Q_{LOO}^{2}$ = 0.6287, *RMSE*_cv_ = 0.7940, $Q_{LMO}^{2}$ = 0.6338, $R_{Y\mathrm{scr}}^{2}$ = 0.1139, $Q_{Y\mathrm{scr}}^{2}$ = −0.2471	*n*_test_ = 9, *RMSE*_ext_ = 0.5570, $R_{\mathrm{ext}}^{2}$ = 0.8232, $Q_{F1}^{2}$ = 0.7482, $Q_{F2}^{2}$ = 0.7228, $Q_{F3}^{2}$ = 0.8173, *CCC* = 0.8781, ${\bar{r}}_{m}^{2}$ = 0.7541, $\Delta r_{m}^{2}$ = 0.0264	*k* = 0.8805, *k* ’= 1.0974, $R_{0}^{2}$ = 0.8132, ${R^{\prime }}_{0}^{2}$ = 0.8186
DNA-	pEC_IR1.5_ = 0.9105 + 0.3091Sv + 0.0493 P_VSA_LogP_7 + 0.2008 Mor03s − 1.0911 T(N..I)	*n*_tr_ = 36, *R*^2^ = 0.7164, $R_{\mathrm{adj}}^{2}$ = 0.6786, *RMSE*_tr_ = 0.6540, *F* = 18.9496	$Q_{LOO}^{2}$ = 0.6221, *RMSE*_cv_ = 0.7550, $Q_{LMO}^{2}$ = 0.5291, $R_{Y\mathrm{scr}}^{2}$ = 0.1200, $Q_{Y\mathrm{scr}}^{2}$ = −0.2504	*n*_test_ = 9, *RMSE*_ext_ = 0.4991, $R_{\mathrm{ext}}^{2}$ = 0.7774, $Q_{F1}^{2}$ = 0.7505, $Q_{F2}^{2}$ = 0.7500, $Q_{F3}^{2}$ = 0.8348, *CCC* = 0.8787, ${\bar{r}}_{m}^{2}$ = 0.6920, $\Delta r_{m}^{2}$ = 0.0076	*k* = 0.9538, *k*’ = 1.0145, $R_{0}^{2}$ = 0.7643, ${R^{\prime }}_{0}^{2}$ = 0.7664

^a^ SpDiam_B(m): spectral diameter from Burden matrix weighted by mass; AVS_B(v): average vertex sum from Burden matrix weighted by van der Waals volume; Eig05_AEA(dm): eigenvalue no. 5 from augmented edge adjacency mat weighted by dipole moment; SddsN: sum of ddsN E-states; Sv: sum of atomic van der Waals volumes (scaled on carbon atom); C%: percentage of C atoms; P_VSA_LogP_7: P_VSA-like on LogP, bin 7; P_VSA_LogP_8: P_VSA-like on LogP, bin 8; T(N..Br): sum of topological distances between N..Br; T(N..I): sum of topological distances between N..I; Mor04s: signal 04/weighted by I-state; Mor03s: signal 03/weighted by I-state; ^b^ *n*_tr_: the number of samples in training set; *R*^2^: coefficient of determination; $R_{\mathrm{adj}}^{2}$: adjusted *R*^2^; *RMSE*_tr_: root mean square error in fitting; *F*: F-value; ^c^ $Q_{LOO}^{2}$: explained variance in leave-one-out prediction; *RMSE*_cv_: root mean square error in cross-validation prediction; $Q_{LMO}^{2}$: explained variance in leave-many-out prediction; $R_{Y\mathrm{scr}}^{2}$ and $Q_{Y\mathrm{scr}}^{2}$: *R*^2^ and *Q*^2^ in Y-scrambling, respectively; ^d^ *n*_test_: the number of samples in test set; *RMSE*_ext_: root mean square error in test set; $R_{\mathrm{ext}}^{2}$: external determination coefficient; $Q_{F1}^{2}$, $Q_{F2}^{2}$ and $Q_{F3}^{2}$: variance explained in test set; *CCC*: concordance correlation coefficient; ${\bar{r}}_{m}^{2}$ and $\Delta r_{m}^{2}$: average and delta $r_{m}^{2}$ values of Roy criteria, respectively; ^e^ *k* and *k*’: slopes of the regression line over external data; $R_{0}^{2}$ and ${R^{\prime }}_{0}^{2}$: *R*^2^ values in Golbraikh & Tropsha criteria.

## Supplementary Materials

Supplementary Materials are available online.
